# The association between psychopathology and substance use: adolescent and young adult substance users in inpatient treatment in Cape Town, South Africa

**DOI:** 10.11694/pamj.supp.2014.17.1.3044

**Published:** 2014-01-18

**Authors:** Amina Saban, Alan Flisher, Ria Laubscher, Leslie London, Neo Morojele

**Affiliations:** 1 Adolescent Health and Research Unit, Department of Psychiatry and Mental Health, University of Cape Town, Cape Town, South Africa; 2 Biostatistics Unit, Medical Research Council, Cape Town, South Africa; 3 School of Public Health and Family Medicine, University of Cape Town, South Africa; 4 Alcohol and Drug Abuse Research Unit, Medical Research Council, Pretoria, South Africa; † Deceased

**Keywords:** Comorbidity, psychopathology, substance use treatment

## Abstract

**Introduction:**

Evidence suggests that comorbid psychopathology can negatively affect treatment outcomes in substance users. In South Africa, limited information exists regarding the prevalence, nature and role of psychiatric comorbidity in substance users. This study examined psychiatric comorbidity and its association with specific substance use, and young adult substance users in treatment for substance use.

**Methods:**

Male and female inpatient substance users (n=95; ages 17-30 years) were sampled consecutively in order of admission from three clinics in Cape Town. An interview schedule was administered to elicit patients’ sociodemographic and substance use history details. The computer-assisted Diagnostic Interview Schedule DSM IV (C-DIS IV) was administered to screen patients for current psychiatric disorders.

**Resuls:**

The sample was largely male, Coloured, Muslim and single. Cannabis (51.6%) and crystal methamphetamine (17.9%) were the most common first substances of use. Heroin (53.7%) and crystal methamphetamine (33.7%) were the most common substances for which treatment was sought (primary substances). The most common comorbid psychopathologies were anti-social personality disorder (ASPD 87.4%) and conduct disorder (CD 67.4%). Regression analyses showed a marginally significant association between specific phobia and first use of cannabis, but indicated no statistically significant associations between psychopathology and substance use.

**Conclusion:**

The results demonstrated a high proportion of previously unidentified comorbid psychopathology in inpatient substance users. Further research is needed to investigate psychiatric comorbidity in inpatient substance users.

## Introduction

In psychiatry, non-substance use psychopathology and problematic substance use are a common form of comorbidity [1]. The comorbid conditions may co-exist simultaneously (concurrent), in tandem (sequential) or separately at any time in the patient's life (lifetime) [2]. Comorbidity is sometimes referred to as ‘dual diagnosis’ or ‘co-occurring disorders’, and can involve the co-occurrence of two or more disorders. [3, 4]. The comorbidity might involve pathology that meets the criteria for diagnosis of a disorder, or might refer to the presence of symptoms of a disorder [5].

Various suggestions have been proposed to explain relationships between comorbid psychopathology and substance. These include i) that people with psychiatric illness attempt to alleviate their discomfort by self-medicating with substances, leading to problematic use of the substances or to substance use-related disorders [2], ii) that the substance use could lead to mental illness [2], iii) that certain individuals might be genetically predisposed to either psychiatric illness and/or substance use resulting in comorbid conditions [2], iv) that either condition could influence, or effect a change in, the course of the other [2], and v) that the substance use and psychopathology share a common neural substrate [4].

Community and hospital-based studies have provided evidence for an increased likelihood of comorbid psychiatric disorder in substance users [5], with a greater likelihood of such comorbidity as the severity of the substance use increases [6]. Psychological and psychiatric problems associated with substance use include cognitive impairment, poor scholastic performance, personal and relationship problems [7], depression, anxiety [8] and PTSD [9]. Disruptive behaviour disorders like antisocial personality disorder [10] and conduct disorder [11] have been found to be very common in patients who receive treatment for substance use [12].

However, the evidence for associations between specific forms of psychopathology and the use of specific substances has not been clear or consistent. For example, the review of community studies by Armstrong and Costello [6] indicated that, except for cannabis, associations between psychiatric disorders and the use of specific substances was not specific to either the psychiatric disorders or the substances of use. In the National Comorbidity Survey (NCS), anxiety disorders and conduct disorder/adult anti-social behaviour disorder were found to precede and predict later alcohol dependence, but such consistency in chronicity was not duplicated with the same certainty with regards to other mental disorders or substances of use [13].

In treatment populations, co-morbidity is known to be characterised by heterogeneity [14]. For example, co-occurring substance use and psychopathology differ with respect to the types of substances and psychiatric problems, the temporality of the comorbid conditions [14], and the possible influence of socio-demographic factors such as age [15], gender or race/ethnicity in the associations [6].

Comorbid substance use and psychiatric disorder has also been associated with poor treatment outcomes for either or both conditions, including increase in substance use and psychosocial impairment, compared with outcomes of treatment when either substance use or psychiatric disorder occur individually [5, 16]. The diagnosis of psychiatric comorbidity in substance users, and of substance use disorders in psychiatric patients, is thus an important component in the development of strategies for treatment [17].

More information is available on comorbid psychiatric disorders and substance use in adults than in children, adolescents and young adults [6, 18]. In South Africa, the prevalence of substance use is a cause for concern, with an increase in the use of substances such as crystal methamphetamine (locally known as ‘tik’) and diacetyl morphine (heroin) [19], and evidence for increased use of substances in general, and cannabis in particular, in adolescents and young adults [20]. Globally, substance use and psychiatric disorders are managed largely independently. Reasons for this include treatment facilities being historically specialised as either substance use treatment centres, or centres for the treatment of psychiatric disorders, with limited numbers of suitably-trained professionals to treat comorbidity [21]. The trend is similar in South Africa. Consequently, patients with comorbid psychopathology and problematic substance use usually attend either a psychiatric or a substance use treatment facility depending on the problem deemed by the patient or his/her family as the one most urgently in need of attention.

In many substance use treatment centres comorbid psychopathology might be suspected in patients, but not assessed or addressed because of a lack of expertise, capacity, time or opportunity at the treatment centres [21]. Consequently, if the substance use treatment results in the patient behaving more socially-appropriately, concurrent psychopathology will receive little attention unless the patient's behaviour becomes disruptive or dysfunctional. Since these behavioural problems are often accompanied by substance use [22], the cycle can be repeated endlessly. Individuals with comorbid psychopathology and substance use can thus repeatedly enter and exit either psychopathology and/or substance use treatment depending on which problem is regarded as the “most problematic” at any one time.

The examination of comorbid psychopathology and substance use in both clinical and community populations of substance users is important particularly because these groups may differ with respect to the nature of the comorbidity [6]. For example, morbidity might be more severe, and comorbidity rates might be higher, in clinical samples compared with community samples. Patients might be more likely to seek treatment for certain disorders (for example, disruptive behaviour disorders) than for others (for example, depression), resulting in clinical samples having a predominance of disorders that are more likely to precipitate admission to treatment [6]. Results from clinical samples might thus not be generalisable to community samples and, conversely, the information obtained from community samples might not apply to clinical populations. Examination of both community and clinical samples is, therefore, needed to ascertain the prevalence of comorbidity in general, and to determine the likelihood of associations between co-morbid conditions.

Information from the South African Stress and Health (SASH) study provides evidence for high prevalence rates of mental disorders in particularly the urban areas of South Africa [22]. However, there has been only limited investigation of the occurrence and nature of comorbid psychiatric diagnoses in substance users in South Africa, and the factors that impact on this comorbidity, for both community [20] and treatment samples [23] of substance users.

This study aimed to assess the frequency and nature of non-substance psychopathology in adolescent and young adult substance users who were receiving inpatient treatment for their substance use, and to examine the association between psychopathology and substance use in these patients, adjusting for social and demographic factors.

## Methods

**Sample:** Ninety-five inpatient substance users were sampled from three privately-funded inpatient substance use treatment centres in Cape Town, South Africa. The treatment centres were selected from the list of Cape Town substance use inpatient treatment centres affiliated to the South African Community Epidemiology Network on Drug Use (SACENDU) [23].The inpatient treatment centres that had the largest number of admissions over the previous six months were shortlisted as potential study sites. Since the Cape Town area is geographically still largely divided in terms of racially classified social groups (RCSGs, as defined by the Population Registration Act of 1950, and consisting of the categories White, Coloured, Black and Indian/Asian) and economic class, three clinic study sites were selected (from the shortlist of treatment centres) from three different suburbs of Cape Town, in an attempt to gain information from as broad a racial and economic spectrum of inpatients within the research period. These were i) a predominantly White upmarket residential-cum-commercial area, and followed a medical model of treatment ii) a middle-class residential area of largely White, Coloured and Indian communities, and followed a treatment modality that included homeopathy, spirituality and Ayurvedic medicine, and iii) an area which included largely Coloured and Black communities, brick homes, informal dwellings, smallholdings and farmland, and provided custodial care, and encouraged spirituality, accompanied by administration of vitamins, massage, periods in a sauna, motivational talks and group therapy.

Sampling of patients was completed over the period December 2008 to December 2009. A minimum sample size of 46 would be required for logistic regression analyses with 6 predictor variables (excluding the constant), a precision of 0.05, 80% power, and a large effect (f2=0.35 or model r2=0.26), while a minimal sample size of 97 would be required for a medium anticipated effect (f2=0.15, or model r2=0.13). We assumed the latter effect being most likely in this study, given previous findings in the literature.A total of 95 young people, constituting all admissions aged 30 years and younger, and admitted for inpatient treatment of problematic substance use.

**Instruments:** An Interview Schedule was used to elicit demographic, social, substance use history and recent substance use information. Demographic and social information included age, gender, racially classified social group (RCSG), religious denomination, highest educational level, referral source, marital status, living arrangements and employment status. Substance use information included age of onset, the first substance of use (other than tobacco), the most frequently used substance, the substance for which treatment was sought, the frequency and quantity of substance use, and previous treatment for substance use. The most frequently used substance (the primary substance of use) was invariably the substance for which treatment was sought.

The computer-assisted Diagnostic Interview Schedule (DIS) for Diagnostic and Statistical Manual (DSM) IV (C-DIS IV) [24] was administered to screen for any current (12-month) psychiatric disorders. Although the C-DIS had not been standardized for a South African population because of the absence of suitable benchmarks against which to measure the instrument, it is one of few available recognized diagnostic instruments that are regarded as sufficiently reliable for use by lay interviewers [25], that shows high concordance with clinically-derived diagnoses [25], and provides the opportunity to screen for current diagnoses when the interviewing time is limited [24]. The C-DIS can be administered by trained, non-clinically experienced examiners, and does not need corroborating details from alternative sources, such as hospital records, to make diagnoses. The C-DIS is considered to be more accurate than the pencil-and-paper version because it automatically counts symptoms for diagnostic criteria, checks dates to ensure accuracy of onset and remission of symptoms. The C-DIS can be used in both treatment and community settings, has the option of being used in either a limited screening version or full version, with the screen version providing information about the presence or absence of a disorder without details regarding the symptoms, course or severity of the disorder.

The full versions of the instrument are recommended to assess disorders with early onset (such as attention deficit hyperactivity disorder, separation anxiety, oppositional disorder and conduct disorder) since these disorders might be risk factors for disorders of later onset. However, the study team felt that the study would not be compromised by use of the screening version with respect to early onset diagnoses because the study sample consisted largely of young adults. To ensure parity in the mode of administration throughout the study, the screened version of the instrument was used for all interviews. All the interviews were conducted by a trained DIS interviewer (primary author).

**Procedure:** Potential study participants were approached after completing a detoxification programme (a period ranging from one to two weeks) offered at each clinic. This was done to ensure that patients had largely overcome the discomfort and agitation associated with withdrawal from substance use, and were more amenable to interviewing. This delay also allowed for the symptoms of substance-induced psychiatric symptoms to be minimized, where present. Each potential study participant was approached to obtain written informed consent. One patient under the age of 18 completed assent forms, and written parental consent was obtained before interviewing this patient. All interviews were conducted by the primary author, at the clinics, and in private. Each interview was completed in one session, with the duration of each session approximating 90 to 120 minutes. The study was approved by the Research Ethics Committee of the University of Cape Town.

### Data analyses

The data were analysed using STATA Version 10 [26]. Percentages were estimated for demographic factors, substance use, and psychiatric diagnoses.

Bivariate associations were assessed using Fisher's Exact tests or chi-squared tests to compare the distribution of patients across the clinics socio-demographically by substance use and by current psychopathology. The percentage of psychopathology and first and primary substances of use and the percentage of first and primary substance use with respect to the most commonly-occurring forms of psychopathology were calculated. Bivariate associations between psychopathology and substance use were calculated using Fisher's Exact and chi-squared analyses. Multiple logistic regression analyses were conducted to determine associations between type of psychiatric disorder (selected from the most commonly-occurring psychiatric diagnoses) and a) the first substances of use, and b) the most frequently used (primary) substances. First substance of use was coded as either cannabis, crystal methamphetamine, or other, based on the most common substances first used; most common primary substance of use was coded as crystal methamphetamine, heroin or other, based on the most common substances for which patients were admitted for treatment. For these, odds ratios and 95% confidence intervals were calculated, unadjusted and adjusted for the treatment centres, and socio-demographic factors including age, gender, religious denomination, racially classified social group and treatment centre. In all the relevant analyses, substance use (in the form of first and primary substances of use) was the dependent variable, and the socio-demographic characteristics and psychopathology diagnoses were the independent variables. Forced statistical modelling was used for the logistic regression.

## Results

Table 1 summarises the characteristics of the sample at the three treatment centres. The total sample consisted of 95 inpatients (ages 17-30 years, with a mean age of 23 years (SD=2.9)). The sample was predominantly male (89.5%), Coloured (88.4%), and Muslim (68.4%). Eighty-six percent of the patients had some secondary school education, 77.9% had never been married, 91.6% lived with immediate family, and 61.1% had entered treatment on their own volition. More than half the sample (54.7%) was unemployed, while nearly a third had fulltime employment, at the time of entering treatment. The proportions of patients at the three clinics differed with respect to two demographic variables - religious denomination (p <0.001), with Clinics 2 and 3 having 90% and 75% Muslim patients respectively, while Clinic 1 had 88.2% Christian patients, and RCSG (p=0.066), with Clinics 2 and 3 having more than 90% Coloured patients while 17.7% of the patients at Clinic 1 were White.

**Table 1:** Demographic, behavioural and psychopathological characteristics of study participant in the three treatment centres (n; %)

	Clinics				
	Clinic 1	Clinic 2	Clinic 3	Total	p-value
	n =17	n=30	n=48	n=95	
Mean Age (SD)	21.7 (3.5)	23.2 (3.0)	23.4 (2.6)	23.0 (2.9)	0.111†
Gender Male	16 (94.1)	24 (80.0)	45 (93.8)	85 (89.5)	0.151††
Racially Classified Social Group					
White	3 (17.7)	1 (3.3)	0	4 (4.2)	0.066††
Coloured	13 (76.5)	27 (90.0)	44 (91.7)	84 (88.4)	
Indian	1 (5.9)	2 (6.7)	4 (8.3)	7 (7.4)	
Religion					
Muslim	2 (11.8)	27 (90.0)	36 (75.0)	65 (68.4)	<0.001††
Christian	15 (88.2)	3 (10.0)	11 (22.9)	29 (30.5)	
None	0	0	1 (1.1)		
Highest educational level					
Primary school	1 (5.9)	0	2 (4.2)	3 (3.2)	0.176††
Secondary school	16 (94.1)	24 (80.0)	42 (87.5)	82 (86.3)	
Tertiary	0	6 (20.0)	4 (8.3)	10 (10.5)	
Referral source					
Self	10 (58.8)	18 (60.0)	30 (62.5)	58 (61.1)	0.250††
Family	3 (17.6)	11 (36.7)	13 (27.1)	27 (28.4)	
Other	4 (23.5)	1 (3.3)	5 (10.4)	10 (10.5)	
Marital status					
Never married	15 (88.2)	22 (73.3)	37 (77.1)	74 (77.9)	0.488††
Other	2 (11.8)	8 (25.7)	11 (22.9)	21 (22.1)	
Living arrangements					
Live alone	0	0	2 (4.2)	2 (2.1)	0.819††
Live with immediate family	16 (94.1)	29 (96.7)	42 (87.5)	87 (91.6)	
Other	1 (5.9)	1 (3.3)	4 (8.3)	6 (6.3)	
Employment status					
Unemployed	7 (41.2)	18 (60.0)	27 (56.3)	52 (54.7)	0.186††
Casually employed	1 (5.9)	2 (6.7)	4 (8.3)	7 (7.4)	
Permanently employed	7 (41.2)	7 (23.3)	17 (35.4)	31 (32.6)	
Other	2 (11.8)	3 (10.0)	0	5 (5.3)	
Usual employment					
Professional	0	0	1 (2.1)	1 (1.1)	0.635††
Skilled	6 (35.3)	10 (33.3)	18 (37.5)	34 (35.8)	
Unskilled	4 (23.5)	10 (33.3)	16 (33.3)	30 (31.6)	
None/student/scholar	7 (41.2)	8 (26.7)	8 (16.7)	23 (24.2)	
Other	0	2 (6.7)	5 (10.4)	7 (7.4)	
Age of first substance use (years)					
10 – 14	13 (76.5)	13 (43.3)	23 (47.9)	49 (51.6)	0.127†
15 – 17	3 (17.6)	11 (36.7)	18 (37.5)	32 (33.7)	
18 – 20	0	5 (16.7)	7 (14.6)	12 (12.6)	
21 – 24	1 (5.9)	1 (3.3)	0	2 (2.1)	
Mean (SD)	13.6 (2.4)	15.1 (2.6)	14.8 (2.3)	14.7 (2.4)	
First substance of use					
Alcohol	4 (23.5)	0	3 (6.3)	7 (7.4)	0.163††
Cannabis	8 (47.1)	16 (53.3)	25 (52.1)	49 (51.6)	
Ecstasy	0	5 (16.7)	4 (8.3)	9 (9.5)	
Heroin	1 (5.9)	2 (6.7)	3 (6.3)	6 (6.3)	
Methaqualone (mandrax)	0	2 (6.7)	0	2 (2.1)	
Crystal methamphetamine (tik)	4 (23.5)	3 (10.0)	10 (20.8)	17 (17.9)	
Multiple	0	2 (6.7)	3 (6.3)	5 (5.3)	
Primary substance of use					<0.001††
Alcohol	3 (17.6)	0	0	3 (3.2)	
Cannabis	4 (23.5)	0	1 (2.1)	5 (5.3)	
Heroin	2 (11.8)	20 (66.7)	29 (60.4)	51 (53.7)	
Crystal methamphetamine (tik)	7 (41.2)	8 (26.7)	17 (35.4)	32 (33.7)	
Methaqualone (mandrax)	0	1 (3.3)	0	1 (1.1)	
Multiple	1 (5.9)	0	1 (2.1)	2 (2.1)	
Frequency of use of primary substance					0.844††
Daily	15 (88.2)	28 (93.3)	43 (89.6)	86 (90.5)	
Few times a week	1 (5.9)	2 (6.7)	3 (6.3)	6 (6.3)	
Sometimes	1 (5.9)	0	2 (4.2)	3 (3.2)	
Intensity/Volume of primary substance use					0.509††
As much as I can obtain	11 (64.7)	14 (46.7)	31 (64.6)	56 (58.9)	
As much as I can afford to buy	6 (35.3)	15 (50.0)	16 (33.3)	37 (38.9)	
Other	0	1 (3.3)	1 (2.1)	2 (2.1)	
Previous treatment for substance use					0.081††
None	11 (64.7)	12 (40.0)	19 (39.6)	42 (44.2)	
Once in past year	3 (17.6)	2 (6.7)	8 (16.7)	13 (13.7)	
More than once in past year	0	5 (16.7)	12 (25.0)	17 (17.9)	
More than a year ago	3 (17.6)	11 (36.7)	9 (18.8)	23 (24.2)	
Age of first treatment for substance use (years)					0.139††
10 – 14	1 (16.7)	2 (11.1)	0	3 (5.7)	
15 – 17	0	4 (22.2)	7 (24.1)	11 (20.8)	
18 – 20	5 (83.3)	7 (38.9)	13 (44.8)	25 (47.2)	
21 – 24	0	3 (16.7)	9 (31.0)	12 (22.6)	
>24	0	2 (11.1)	0	2 (3.8)	
Mean age of first treatment for substance use (SD)	17.5 (3.3)	18.9 (3.9)	19.3 (2.6)	19 (3.1)	0.454†
Psychopathology					
No previous diagnosis of psychopathology	15 (88.2)	29 (96.7)	47 (97.9)	91 (95.8)	0.298
Generalised anxiety disorder	3 (17.6)	1 (3.3)	1 (2.1)	5 (5.3)	0.059
Post-traumatic stress disorder (PTSD)	3 (17.6)	5 (16.7)	6 (12.5)	14 (14.7)	0.065
Depression	6 (35.3)	9 (30.0)	9 (18.8)	24 (25.3)	0.291
Mania	6 (35.3)	1 (3.3)	5 (10.4)	12 (12.6)	0.007
Schizophrenia	0	2 (6.7)	0	2 (2.1)	0.128
Obsessive Compulsive Disorder (OCD)	2 (11.8)	1 (3.3)	1 (2.1)	4 (4.2)	0.298
Eating disorder	0	2 (6.7)	2 (4.2)	4 (4.2)	0.656
Separation anxiety	1 (5.9)	2 (6.7)	2 (4.2)	5 (5.3)	0.848
Oppositional defiant disorder	7 (41.2)	10 (33.3)	15 (31.1)	32 (33.7)	0.771
Conduct disorder	10 (58.8)	18 (60.0)	36 (75.0)	64 (67.4)	0.263
Antisocial personality disorder	14 (82.4)	26 (86.7)	43 (89.6)	83 (87.4)	0.657
Pain disorder	3 (17.7)	4 (13.3)	3 (6.3)	10 (10.5)	0.330
Specific phobia	4 (23.5)	5 (16.7)	6 (12.5)	15 (15.8)	0.565
Substance dependence	12 (70.6)	29 (96.7)	46 (95.8)	87 (91.6)	0.007
Substance abuse	4 (23.5)	5 (16.7)	6 (12.5)	15 (15.8)	0.606

† Comparisons between clinics based on Kruskal-Wallis testing for age, and on chi-squared test for other sociodemographic variables

†† Fisher's Exact testing for expected frequencies <5

Approximately 52% of the sample had commenced substance use between the ages of 10 and 14 years while 85.3% (n=81) had started using substances by age 17 years, with a mean age of substance use onset of 14.7 years (SD=2.4). The most common first substances of use were cannabis (51.6%) and crystal methamphetamine (tik) (17.9%). The most common substances for which treatment was sought (i.e. primary substances of use) were heroin (53.7%) and crystal methamphetamine (33.7%). The large majority of patients used substances every day (90.5%), and used as much as they could obtain (58.9%) or afford to buy (38.9%). All but two participants smoked cigarettes daily, did not count how many cigarettes they smoked, and did not regard their cigarette smoking as a problem (not shown). There was a statistically significant difference in the proportion of patients at the three clinics with respect to the primary substance of use. Heroin was the primary substance of use in more than 60% of the patients at Clinics 2 and 3, compared with 11.8% at Clinic 1. At Clinic 1, more than 40% of the patients had crystal methamphetamine as their primary substance of use compared with 26.7% and 35.4% at Clinics 2 and 3 respectively. Forty-four percent of the patients were in substance use treatment for the first time at the time of the study. Of those who had had previous treatment for their substance use (n=53), 68% (n=36) had been between the ages of 15 and 20 years when they had their first treatment. However, the proportion of patients at each clinic was marginally different with respect to their previous history of substance use treatment (p=0.081), with the majority of patients at Clinic 1 (64.7%) being in substance use treatment for the first time.

Table 2 lists the proportions of patients at each of the clinics with respect to their diagnosis of current psychopathology. In the study all the C-DIS IV modules were administered, but only the most common psychiatric diagnoses are listed in Table 2. Ninety-six percent (Table 1) of patients had some form of psychopathology; only three patients had no non-substance use psychiatric diagnosis. Some patients had more than one current psychiatric disorder. Sixty patients (63.2%) had at least two non-substance use psychopathology diagnoses, and 27 patients (28.4%) had at least three non-substance use psychopathology diagnoses. Of the 92 patients with psychopathology, only four patients had previously been diagnosed with a non-substance use psychiatric disorder (Table 2). There was no statistically significant difference in the proportions of patients at each clinic who had never previously been diagnosed with a psychiatric disorder. The most common current psychiatric diagnoses were substance dependence (91.6%), anti-social personality disorder (ASPD) (87.4%), conduct disorder (CD) (67.4%), oppositional defiant disorder (33.7%) and major depression (25.3%). The proportion of patients differed significantly at the three clinics with respect to mania (p=0.007), with one patient at Clinic 2 having had a manic episode in the last 12 months compared with six and five patients at Clinics 1 and 3 respectively. The numbers of patients diagnosed with substance dependence differed significantly across the treatment centres (p=0.007), with Clinics 2 and 3 having more than 95% of patients dependent on substances compared with 70.6% of patients with substance dependence at Clinic 1. The clinics also differed marginally with respect to generalised anxiety disorder (p=0.059) and post-traumatic stress disorder (PTSD) (p=0.065) although the numbers of patients with these diagnoses were generally relatively small.

**Table 2:** Frequency of most common first and primary substances of use by most common psychopathology diagnoses

	First substance of use			
Psychopathology	Cannabis	p-value†	Crystal Methamphetamine	p-value†
Conduct disorder	38 (59.3)	0.048	11 (17.2)	0.782
Anti-social personality disorder	46 (55.4)	0.065	14 (16.9)	0.445
Major depression	14 (58.3)	0.486	2 (8.3)	0.223
Oppositional defiant disorder	20 (62.5)	0.192	4 (12.5)	0.405
	Most common primary substance of use			
	Heroin	p-value†	Crystal Methamphetamine	p-value†
Conduct disorder	19 (29.7)	0.170	15 (23.4)	0.592
Anti-social personality disorder	29 (34.9)	1.000	16 (19.3)	0.271
Major depression	8 (33.3)	1.000	3 (12.5)	0.385
Oppositional defiant disorder	9 (28.1)	0.371	10 (31.1)	0.111

† Chi-squared testing when expected frequencies >5 and Fisher's Exact testing when expected frequencies <5

There was no statistically significant difference in the proportions of any psychopathology by either first substance of use or by primary substance of use (results not shown). Table 3 presents the percentage of patients with the most common first and primary substances of use in terms of the most commonly-occurring non-substance use psychopathology. These results indicate that a statistically significant proportion of those who were positive for conduct disorder had started out using cannabis (p=0.048) compared with those who were not positive for conduct disorder. The proportion of patients who had anti-social personality disorder and had used cannabis as their first substance was marginally higher than the proportion of patients who were positive for the other commonly-occurring disorders and used cannabis as their first substance of use. The difference in the proportions of the other psychopathologies by substance use were not statistically significant.

**Table 3:** Prevalence of most common substance use by most common psychopathology*

First substance of use n (%)				
Most common psychopathology (n)	Cannabis (Total = 49)	P	Crystal Methamphetamine (Total = 17)	p
**Conduct disorder**				0.796
Present: 64	38 (59.4)	0.049	11 (17.2)	
Absent: 31	11 (35.5)		6 (19.4)	
**Oppositional defiant disorder**				0.405
Present: 32	20 (62.5)	0.193	4 (12.5)	
Absent: 63	29 (46.0)		13 (20.6)	
**Major depression**				0.223
Present: 24	14 (58.3)	0.596	2 (8.3)	
Absent: 71	35 (49.3)		15 (21.1)	
**Anti-social personality disorder**				0.445
Present: 83	46 (55.4)	0.064	14 (16.9)	
Absent: 12	3 (25.0)		3 (25.0)	
**Primary substance of use n (%)**				
Most common psychopathology (n)	Heroin (Total = 33)	p	Crystal Methamphetamine (Total = 20)	p
Conduct disorder				0.587
Present: 64	19 (29.7)	0.209	15 (23.4)	
Absent: 31	14 (45.2)		5 (16.1)	
**Oppositional defiant disorder**				0.141
Present: 32	9 (28.1)	0.461	10 (50.0)	
Absent: 63	24 (38.1)		10 (15.9)	
**Major depression**				0.385
Present: 24	8 (57.1)	0.867	3 (21.4)	
Absent: 71	25 (35.2)		17 (23.9)	
**Anti-social personality disorder**				0.271
Present: 83	29 (34.9)	1.000	16 (34.8)	
Absent: 12	4 (33.3)		4 (33.3)	

* Chi squared tests were used to calculate p-values when cell sizes were >5 and Fisher's Exact was used when cell sizes were <5

Table 2 lists the percentage of the most common first and primary substances of use by the most common psychopathology. Significantly more patients (p=0.049) who were found to be positive for conduct disorder, compared with those who were not diagnosed with conduct disorder, had used cannabis as their first substance. Marginally more patients (p=0.0654) who were found to be positive for antisocial personality disorder, compared with those who were not positive for antisocial personality disorder, had their substance use debut with cannabis.

Table 4 documents the results of the regression analyses which were conducted to determine associations between different forms of non-substance use psychopathology and different forms of first substance used and primary substance use at bivariate level. Anti-social personality disorder was marginally associated with cannabis as the first substance of use (p=0.061). However, this association was no longer significant after adjusting for demographic factors (p=0.117). Similarly, the significant association between conduct disorder and cannabis as the first substance of use (p=0.031) was no longer significant after adjusting for demographic factors (p=0.103).

**Table 4:** Association between psychopathology and substance use (OR, 95% CI and p-value)†

		First substance of use				Most common primary substance of use			
		Cannabis		Crystal methamphetamine		Heroin		Crystal methamphetamine	
Psychopathology		Unadjusted	Adjusted	Unadjusted	Adjusted	Unadjusted	Adjusted	Unadjusted	Adjusted
**Anti-social personality**	**OR 95% CI p-value**	3.730	3.228	0.609	0.644	1.074	1.496	0.478	0.424
		0.942-	0.745-	0.146-	0.136-	0.298-	0.326-	0.128-	0.100-
		14.773	13.975	2.537	3.047	3.872	6.857	1.785	1.800
		0.061	0.117	0.495	0.579	0.913	0.604	0.272	0.245
**Conduct disorder**	**OR 95% CIp-value**	2.657	2.256	0.865	0.722	0.513	0.413	1.592	1.895
		1.092-	0.847-	0.287-	0.213-	0.211-	0.139-	0.520-	0.553-
		6.464	6.006	2.605	2.443	1.246	1.229	4.870	6.493
		0.031	0.103	0.796	0.600	0.140	0.112	0.415	0.309
**Oppositional defiant**	**OR 95% CIp-value**	1.954	1.920	0.549	0.503	0.636	0.670	2.409	2.192
		0.818-	0.749-	0.163-	0.139-	0.253-	0.232-	0.879-	0.752-
		4.666	4.917	1.847	1.824	1.601	1.939	6.599	6.385
		0.131	0.174	0.333	0.296	0.336	0.460	0.087	0.150
**PTSD**	**OR 95% CIp-value**	0.929	1.571	0.313	0.334	2.115	2.207	0.251	0.245
		0.299-	0.388-	0.038-	0.030-	0.672-	0.482-	0.031-	0.025-
		2.888	6.363	2.568	3.753	6.659	10.116	2.045	2.385
		0.898	0.527	0.279	0.375	0.200	0.308	0.197	0.226
**Major depression**	**OR 95% CIp-value**	1.440	1.669	0.339	0.586	0.920	1.014	0.454	0.569
		0.565-	0.537-	0.072-	0.111-	0.346-	0.296-	0.120-	0.131-
		3.670	5.183	1.608	3.083	2.448	3.467	1.710	2.479
		0.445	0.376	0.173	0.528	0.867	0.983	0.243	0.453
**Specific phobia**	**OR 95% CIp-value**	1.500	4.739	0.286	0.417	1.309	1.665	0.926	1.098
		0.488-	0.990-	0.035-	0.045-	0.422-	0.382-	0.235-	0.230-
		4.607	22.677	2.337	3.857	4.060	7.249	3.660	5.237
		0.479	0.051	0.243	0.441	0.641	0.497	0.913	0.906
**Pain disorder**	**OR 95% CI p-value**	1.144	1.077	1.022	1.609	0.675	0.677	1.478	1.812
		0.324-	0.276-	0.200-	0.269-	0.166-	0.124-	0.354-	0.354-
		4.040	4.199	5.221	9.634	2.737	3.699	6.174	9.263
		0.834	0.915	0.979	0.602	0.582	0.652	0.592	0.475

† Adjusted for age, gender, racially classified social group, religion and treatment centre

The unadjusted odds ratio for the association between specific phobia and cannabis as the first substance of use was not statistically significant (p=0.479) but approached statistical significance on adjustment (OR=4.74; 95% CI 0.99-22.66, p=0.051). The association between oppositional defiant disorder and crystal methamphetamine as the primary substance of use was only marginally significant (p=0.087) at bivariate level.

## Discussion

This study examined the frequency and nature of non-substance use psychopathology in young adult substance users in inpatient treatment for their substance use, and to identify demographic, social and substance use factors that influenced the association between psychopathology and substance use.

The results obtained indicate a large proportion of inpatient substance users who had not previously been diagnosed with a psychiatric disorder, while a large number of patients was diagnosed with a current (12-month) non-substance psychiatric disorder in this study, demonstrating a high percentage of comorbid psychopathology in these inpatients in Cape Town, with a percentage that exceeds the prevalence of these psychiatric diagnoses reported for the general adult community but using a different instrument, namely the Composite International Diagnostic Interview (CIDI) which also provides DSM IV diagnoses [22].

The high percentage of previously-undiagnosed psychopathology in these inpatient substance users, despite many of the patients having been in treatment for their problematic substance use previously, suggests a need for substance users to be assessed for co-occurring psychopathology as part of treatment and rehabilitation. It might also be necessary to recognize the demographic and social heterogeneity of these patients, and to tailor their treatment according to their individual needs.

It is likely that the study sample was vulnerable to Berkson's Bias [27] in that there would be an increased likelihood of patients seeking treatment for their substance use problems because they experienced a co-existing non-substance use psychiatric disorder. The presence of disruptive behavior disorders (noted by the high prevalence of diagnoses such as anti-social personality disorder, conduct disorder and oppositional defiant disorder) could also have played a role in treatment-seeking by causing social conflict in the lives of the substance users [7], precipitating admission for substance use treatment. It is also possible that the percentage of psychopathology in this substance use treatment group differed from that in community substance users because of differences in the severity of the extant conditions in the two groups [6]. In other words, substance users in the community might experience symptoms related to the same psychiatric diagnoses as those of patients in this study, but these psychiatric symptoms might be less severe and not yet meet the criteria for diagnoses and hence might not yet play a role in treatment-seeking behavior.

While there is debate on whether substance use (in the form of problematic substance use, or abuse or dependence) may be regarded as a dysfunctional or antisocial behaviour, constituting part of a psychiatric disorder, or a psychiatric disorder itself [8], there is little doubt about the predominance of disruptive behavior disorders in clinical samples of substance users compared with the prevalence of other non-substance use psychopathology [7], and the role that disruptive behaviour disorders might play in treatment-seeking.

The presence of a comorbid disruptive behavior disorder might also be more likely to precipitate treatment- seeking for substance use problems than would a co-occurring anxiety disorder [6], possibly accounting for the low proportions, or absence, in the present study, of those comorbid psychiatric diagnoses (for example, depression [8] anxiety [7] and posttraumatic stress disorder [6]) that have commonly been associated with substance use, abuse or dependence in the community [7, 8] and in patients who receive treatment for their substance use [5].

The proportions of patients with the most common psychiatric diagnoses did not show statistically significant differences by either the first or primary substances of use. However, a significantly greater proportion of patients who were diagnosed with conduct disorder and anti-social personality disorder had also initiated their substance use with cannabis. It is thus possible that cannabis was a notable first substance of use in those who were diagnosed with conduct disorder or antisocial personality disorder. The the marginal results obtained in this study, however, must be interpreted with caution.

There might be other factors that also play a role in the nature, prevalence and associated substance use of psychopathology amongst inpatient substance users. For example, when examining the psychiatric symptoms of patients in the National Treatment Outcome Research Study, Marsden et al. [7], found that the relationship between psychiatric symptoms and substance use was not a direct relationship but rather a relationship that was conditional on the types of substance use. For example, these authors reported that depressive symptoms were less likely or less severe in opiate users in treatment, than in users of stimulants who were in treatment. They also found that, in substance users who receive treatment, the frequency and severity of psychiatric symptoms were predicted by poor physical health, previous psychiatric treatment, gender, and personal relationships characterized by high levels of conflict. It is thus possible that factors such as these, of which physical health, previous psychiatric treatment and personal relationships were not assessed in relation to comorbidity in the present study, contributed to the findings of the present study by influencing psychiatric symptoms and disorders, and, indirectly, the association between psychopathology and substance use.

The distribution of the Western Cape province (of which Cape Town is the capital city) treatment population during the study period, in terms of racially classified social grouping, indicates that Coloured patients who presented for substance use treatment constituted the majority of the treatment population [28]. The study sample thus reflects the population preponderance of Coloured substance use patients relative to White and Indian patients. The complete absence of Black patients from this sample is, however, surprising, particularly considering the close proximity to Clinic 3 of a largely Black informal settlement. This finding could possibly be a result of a combination of factors that involved financial constraints and/or the nature of the treatment offered at the treatment centres selected for sampling.

The sample contained few females. The proportion of males (90%) to is consistent with the gender distribution of the substance using population that sought treatment, from which the sample was drawn [28], and with samples of similar studies [29, 30]. On examining possible reasons for the uneven gender distribution of substance use inpatients, Green et al.^31^ found that in females, a comorbid psychiatric diagnosis predicted a failure of treatment initiation, while in males a low educational level predicted a failure of treatment initiation. It is possible that comorbid psychopathology might have played a role in the treatment initiation of some females, but this association was beyond the design of the present study, as was the role of low educational level in potential male substance use inpatients in treatment initiation.

However, the paucity of females in this study sample could also be indicative of females possibly facing more obstacles to entering inpatient treatment. For example, women may face more social stigma and treatment beliefs [31], or financial constraints [32] compared with males.

Further investigations could be geared at identifying and minimizing the obstacles that females face with regards to attending inpatient treatment facilities. Based on the findings of Green et al. [33], it might thus be useful to emphasise assessment of females for psychiatric disorder prior to suggesting inpatient admission. Such assessment, coupled with treatment for psychiatric disorder and counseling, might aid admission of females where this is deemed appropriate and potentially beneficial. Similar support for males with low educational levels might also prove helpful in aiding initiation of treatment for substance use.

The high percentage of cigarette smoking amongst this group of substance users in treatment might be cause for concern. Most participants viewed their cigarette smoking as more socially acceptable and with a smaller impact on their lives than their use of other substances. Cigarette smokers who receive treatment for use of other substances could potentially face a future of compromised health as a result of their cigarette smoking, irrespective of whether or not they attain abstinence from their other substance use.

It is noteworthy that few of the patients were in treatment for problem-drinking of alcohol when alcohol is still the most common substance of abuse for which treatment is sought in the area [28]. The preponderance of Muslim patients in this sample could have accounted for this result since alcohol consumption is forbidden in Islam and is considered a social taboo in Muslim communities, even among users of other substances. However, alcohol generally appears to be an unlikely substance of use for which treatment is sought in young people, particularly when there are other (usually illicit) substances of use available [34]. The most likely reason for the small number of patients in treatment for alcohol use in this sample might thus be that patients in treatment for alcohol use were usually older [28] and thus not eligible for selection in the study.

The present study did not elicit information regarding the mode of substance use. Injecting substances like heroin is known to be associated with increased Severity of Dependence scores compared with smoking heroin [35], while increased severity of dependence on substances has been associated with increased risk of psychopathology [13]. In the absence of information regarding mode of drug administration, its relevance for associations between substance use, substance use severity and psychopathology cannot be commented on. However, smoking appears to be the most common means of non-alcohol substance administration in this community [28] so it would be appropriate to assume that, where relevant, substances in this sample were smoked rather than injected.

Study findings should be interpreted in light of potential study limitations. Firstly, because of the small sample size, the multiple comparisons could have produced differences between groups that might be chance findings, and real differences between groups might have been missed. Secondly, the lack of a representative sample precludes generalisability of the results beyond the study sample. Lastly, the cross-sectional design of the study limits inferences regarding temporality, causality, and gateway pathways to comorbid substance use and psychopathology, even in those cases where statistically significant associations emerged. Further investigations in the form of longitudinal studies that assess the risk for substance use in individuals diagnosed with psychiatric disorder and the risk for psychiatric disorder in substance users are needed to provide information regarding temporal associations between the psychopathology and substance use. Further investigations of both treatment and community samples might provide additional insights, particularly as regards differences in comorbid non-substance use psychopathology.

## Conclusion

This study has highlighted that psychopathology is common in substance using young people who receive inpatient treatment for their substance use. The study has illustrated the need for psychiatric assessment of comorbid psychopathology in substance users who receive treatment for their substance use in Cape Town, South Africa, with the suggestion that integrated service models be developed for the treatment of mental illness and substance use.

## References

[1] Volkow N (2001). Drug abuse and mental illness: progress in understanding comorbidity. Am J Psychiatry..

[2] Bukstein OG, Brent DA, Kaminer Y (1989). Comorbidity of substance abuse and other psychiatric disorders in adolescents. Am J Psychiat..

[3] Chambers RA (2008). What's in a name: ‘Dual Diagnosis’ vs ‘Co-occurring Disorders’. J Dual Diag..

[4] Chambers RA (2008). What's in a name: “Triple Diagnosis”. J Dual Diag..

[5] Schuckit MA (2006). Comorbidity between substance use disorders and psychiatric conditions. Addiction..

[6] Armstrong TD, Costello EJ (2002). Community studies on adolescent substance use, abuse or dependence, and psychiatric comorbidity. J Consult Clin Psych..

[7] Marsden J, Gossop M, Stewart D, Rolfe A, Farrell M (2000). Psychiatric symptoms among clients seeking treatment for drug dependence. Brit J Psychiat..

[8] Rey JM, Martin A, Krabman P (2004). Is the party over? Cannabis and juvenile psychiatric disorder: the past 10 years. J Am Acad Child AdolescPsychiat..

[9] Triffleman EG, Marmar CR, Delucchi KL, Ronfeldt H (1995). Childhood trauma and posttraumatic stress disorder in substance abuse inpatients. J Nerv Ment Dis..

[10] Gerstley LJ, Alterman M, McClellan AT, Woody GE (1990). Antisocial Personality Disorder in patients with substance usedisorders: a problematic diagnosis?. Am J Psychiat..

[11] Couwenbergh C, van den Brink W, Zwart K, Vreugdenhil C, Van Wijngaarden-Cremers P, van der Gaag RJ (2006). Comorbid psychopathology in adolescents and young adults treated for substance use disorders: a review. Eur Child AdolescPsychiat..

[12] Grilo CM, Becker DF, Fehon DC, Edell WS, McGlashan TH (1996). Conduct disorder, substance use disorders and coexisting conduct and substance use disorders in adolescent inpatients. Am J Psychiat..

[13] Kessler RC, Nelson CB, McGonagle KA, Edlund MJ, Frank RG, Leaf PJ (1996). The epidemiology of co-occurring addictive and mental disorders: implications for prevention and service utilisation. Am J of Orthopsychiatr..

[14] Lehman AF, Myers CP, Thompson JW, Corty E (1993). Implications of mental and substance use disorders: a comparison of single and dual diagnosis patients. J Nerv Ment Dis..

[15] Bird HR, Gould MS, Staghezza BM (1993). Patterns of diagnostic comorbidity in a community sample of children aged 9 through 16 years. J Am Acad Child AdolescPsychiat..

[16] Ciraulo DA, Piechniczek-Buczek J, Iscan EN (2003). Outcome predictors in substance use disorders. PsychiatrClin North Am..

[17] Landheim AS, Bakken K, Vaglum P (2006). Impact of comorbid psychiatric disorders on the outcome of substance users: a six-year prospective follow-up in two Norwegian counties. BMC Psychiatry..

[18] Saban A, Flisher AJ (2010). The association between psychopathology and substance use: a review of the literature. J Psychoactive Drugs..

[19] Weich L, Perkel C, van Zyl N, Rataemane S, Naidoo L (2010). South African guidelines for the management of opioid dependence: updated 2009. S Afr Med J..

[20] Van Heerden MS, Grimsrud AT, Seedat S, Myer L, Williams DR, Stein DJ (2009). Patterns of substance use in South Africa: results from the South African Stress and Health study. S Afr Med J..

[21] Kaminer Y, Connor DF, Curry JF (2007). Comorbid adolescent substance use and major depressive disorders: a review. Psychiatry..

[22] Herman AA, Stein DJ, Seedat S, Heeringa SG, Moomal H, Williams DR (2009). The South African Stress and Health (SASH) study: 12-month and lifetime prevalence of common mental disorders. S Afr Med J..

[23] Pluddemann A, Parry C, Bhana A, Dada S, Fourie D (2009). South African Community Epidemiology Network on Drug Use (SACENDU) Update. http://www.sahealthinfo.org/admodule/sacendu/report1cover.pdf.

[24] Robins LN, Cottler LB, Bucholz KK, Compton WM, North CS, Rourke KM (2002). Diagnostic Interview Schedule for DSM IV.

[25] Helzer JE, Robins LN, McEvoy LT, Spitznagel EL, Stoltzman RK, Farmer A, Brockington IF (1985). A comparison of clinical and Diagnostic Interview Schedule diagnoses. Arch Gen Psychiat..

[26] STATA Version 10 (2007).

[27] DuFort GG, Newman SC, Bland RC (1993). Psychiatric comorbidity and treatment-seeking: sources of selection bias in the study of clinical populations. J Nerv Ment Dis..

[28] Pluddemann A, Dada S, Parry C, Bhana A, Perreira T, Nel E, Mncwabe T, Gerber W, Aboagye L (2010). Monitoring alcohol and drug abuse trends in South Africa (July 1996-December 2009. SACENDU Research Brief..

[29] Greenfield SF, Brooks AJ, Gordon SM, Green CA, Kropp F, McHugh RK, Lincoln M, Hien D, Miele GM (2007). Substance use treatment entry, retention and outcome in women: a review of the literature. Drug Alcohol Depend..

[30] Tuchman E (2010). Women and addiction: the importance of gender issues in substance use research. J Addict Dis..

[31] Myers B, Fakier N, Louw J (2009). Stigma, treatment beliefs, and substance abuse treatment use in historically disadvantaged communities. Afr J Psychiatry..

[32] Myers BJ, Louw J, Pasche SC (2010). Inequitable access to substance abuse treatment services in Cape Town, South Africa. http://www.biomedcentral.com/content/pdf/1747-597X-5-28.pdf.

[33] Green CA, Polen MR, Dickinson DM, Flynch FL, Bennett MD (2002). Gender differences in predictors of initiation, retention and completion in an HMO-based substance abuse treatment program. J Subst Abuse Treat..

[34] Brown SA, Cleghorn A, Schuckit MA, Myers MG, Mott MA (1996). Conduct disorder among adolescent alcohol and drug abusers. J Stud Alcohol..

[35] Strang J, Griffiths P, Powis B, Gossop M (1999). Heroin chasers and heroin injectors: differences observed in a community sample in London, UK. Am J Addict..

